# Sustained delivery of celecoxib from nanoparticles embedded in hydrogel injected into the biopsy cavity to prevent biopsy-induced breast cancer metastasis

**DOI:** 10.1007/s10549-024-07410-x

**Published:** 2024-07-05

**Authors:** Reese Simmons, Hiroyasu Kameyama, Seiko Kubota, Yunguang Sun, John F. Langenheim, Rana Ajeeb, Tristan S. Shao, Samantha Ricketts, Anand C. Annan, Natalie Stratemeier, Sophie J. Williams, John R. Clegg, Kar-Ming Fung, Inna Chervoneva, Hallgeir Rui, Takemi Tanaka

**Affiliations:** 1 Stephenson Cancer Center, University of Oklahoma Health Sciences Center, Oklahoma City, OK 73104 USA; 2 Department of Pathology, Medical College of Wisconsin, Milwaukee, WI 53226 USA; 3 Department of Pharmacology, Physiology & Cancer Biology, Sidney Kimmel Cancer Center, Thomas Jefferson University, Philadelphia, PA 19107 USA; 4 Stephenson School of Biomedical Engineering, University of Oklahoma, Norman, OK 73019 USA; 5 Department of Pathology, School of Medicine, University of Oklahoma Health Sciences Center, Oklahoma City, OK 73104 USA; 6 Department of Radiological Sciences, School of Medicine, University of Oklahoma Health Sciences Center, Oklahoma City, OK 73104 USA; 7 Institute for Biomedical Engineering, Science, and Technology, University of Oklahoma, Norman, OK 73019 USA; 8 Division of Biostatistics, Department of Pharmacology, Physiology & Cancer Biology, Sidney Kimmel Cancer Center, Thomas Jefferson University, Philadelphia, PA 19107 USA; 9 Department of Pathology, Stephenson Cancer Center, School of Medicine, University of Oklahoma Health Sciences Center, 975 NE 10th St, BRC-W, Rm 1415, Oklahoma City, OK 73104 USA

**Keywords:** Hydrogel, Nanoparticle, Metastasis, Biopsy, Biopsy site marker, Local drug delivery

## Abstract

**Purpose:**

We have previously reported that protracted Cyclooxygenase-2 (COX-2) activity in bone marrow-derived cells (BMDCs) infiltrating into biopsy wounds adjacent to the biopsy cavity of breast tumors in mice promotes M2-shift of macrophages and pro-metastatic changes in cancer cells, effects which were suppressed by oral administration of COX-2 inhibitors. Thus, local control of COX-2 activity in the biopsy wound may mitigate biopsy-induced pro-metastatic changes.

**Methods:**

A combinatorial delivery system—thermosensitive biodegradable poly(lactic acid) hydrogel (PLA-gel) incorporating celecoxib-encapsulated poly(lactic-co-glycolic acid) nanoparticles (Cx-NP/PLA-gel)—was injected into the biopsy cavity of Py230 murine breast tumors to achieve local control of COX-2 activity in the wound stroma.

**Results:**

A single intra-biopsy cavity injection of PLA-gel loaded with rhodamine-encapsulated nanoparticles (NPs) showed sustained local delivery of rhodamine preferentially to infiltrating BMDCs with minimal to no rhodamine uptake by the reticuloendothelial organs in mice. Moreover, significant reductions in M2-like macrophage density, cancer cell epithelial-to-mesenchymal transition, and blood vessel density were observed in response to a single intra-biopsy cavity injection of Cx-NP/PLA-gel compared to PLA-gel loaded with NPs containing no payload. Accordingly, intra-biopsy cavity injection of Cx-NP/PLA-gel led to significantly fewer metastatic cells in the lungs than control-treated mice.

**Conclusion:**

This study provides evidence for the feasibility of sustained, local delivery of payload preferential to BMDCs in the wound stroma adjacent to the biopsy cavity using a combinatorial delivery system to reduce localized inflammation and effectively mitigate breast cancer cell dissemination.

**Supplementary Information:**

The online version contains supplementary material available at 10.1007/s10549-024-07410-x.

## Introduction

Breast cancer is the most commonly diagnosed malignancy in the United States [1]. Despite over 60% of breast cancer cases being diagnosed at an early stage, the recurrence rate remains as high as 30% [2–4]. Mounting evidence suggests that delaying surgery after diagnosis is associated with an increased risk of disease progression [5] and mortality [6] in early-stage breast cancer patients [5–9]. Accordingly, in 2022, the Commission on Cancer recommended timely surgery within 60 days of diagnosis as a part of quality measures [10]. However, given the clinical options patients may benefit from during the interval between diagnosis and surgery and/or logistical issues (e.g., multi-modal imaging, molecular tumor profiling, genetic testing, egg preservation, a second opinion, scheduling of multidisciplinary surgery, etc.), resection of the tumor within the 60-day window recommendation may not always be achievable. In fact, the interval between breast cancer diagnosis and surgery has grown substantially over the past two decades [11]. Thus, it becomes critical to implement a prevention strategy considering the biological mechanism(s) underlying the disease progression and mortality that results from delaying surgery.

We have previously reported a sustained accumulation of macrophages (Mϕ) around the needle biopsy site in surgically resected early-stage breast tumors (*n* = 73) [12]. In preclinical mouse models, we found that needle biopsy of breast tumors promoted epithelial-to-mesenchymal transition (EMT) and pulmonary metastasis through paracrine factors pertinent to M2-like Mϕ [13]. Additionally, Cyclooxygenase-2 (COX-2) activity was primarily induced in BMDCs in the wound stroma adjacent to the biopsy cavity, leading to protracted inflammation during the interval between biopsy and surgery, which underlies biopsy-induced pro-metastatic changes [13]. Accordingly, oral administration of celecoxib, which blocks COX-2 activity, effectively mitigated needle biopsy-induced M2ϕ accumulation, cancer cell EMT, angiogenesis, and pulmonary metastasis [13]. Celecoxib is one of a class of non-steroidal anti-inflammatory drugs (NSAIDs) that selectively inhibits COX-2 activity and is indicated for rheumatoid arthritis, osteoarthritis, and acute pain management [14]. While needle biopsy instigates local inflammation as a part of the wound healing process [12, 15–17], pharmacological management of post-biopsy inflammation is not currently standardized; the use of an ice pad or over-the-counter painkillers, such as acetaminophen, is generally recommended [18]. Prolonged oral administration of NSAIDs is neither practical nor recommended [18, 19], based on the potential risk of systemic adverse effects that may outweigh the benefit.

Local drug delivery systems have emerged as highly effective in treating multiple pathologies, including various cancers [20–22]. Local delivery of therapeutic payload enables enhanced efficacy when an effective dose is selectively delivered to the area of interest while also minimizing systemic toxicity due to off-target effects. The Gliadel® (Eisai Inc., Nutley, NJ, USA) wafer is one successful example of such a drug delivery strategy, in which the alkylating chemotherapeutic payload (carmustine) is locally delivered for approximately three weeks in malignant glioma by insertion into the open cavity following surgical resection of the tumor [23, 24]. Because the cytotoxicity of carmustine depends on its dose and exposure time [25], local delivery allows for long-term release of carmustine, improving therapeutic efficacy and patient quality of life [26].

In the clinical setting, immediately after tissue sampling, a biopsy-site marker, composed of a small radio-opaque metal clip coated with biomaterials, is placed into the biopsy cavity to help surgeons identify the location of carcinomas at the time of surgery. Different types of biocompatible and biodegradable coating materials have been adopted to prevent the dislocation of metal clips within the cavity [27]. Yet they have not been explored as a drug delivery carrier of therapeutics within the biopsy cavity.

We rationalized that local delivery of celecoxib directly into the biopsy cavity would be preferentially delivered to BMDCs due to their proximity, and thereby reduce the risk of COX-2 mediated, biopsy-associated pro-metastatic changes and subsequent metastasis. Thus, we investigated whether injection of a biodegradable, thermosensitive poly(lactic acid) hydrogel (PLA-gel) loaded with celecoxib-encapsulated poly(lactic-co-glycolic acid) nanoparticles (PLGA-NPs) into the biopsy cavity of breast tumors would prevent biopsy-induced inflammation, EMT, M2ϕ accumulation, angiogenesis, and pulmonary metastasis in a mouse model of breast cancer.

## Methods

### PLGA-nanoparticle preparation

PLGA-NPs were prepared following the nanoprecipitation technique with 40 mg of Resomer 502H (PLGA *M*_*w*_ = 7–17 kDa, 50:50 L:G ratio; Sigma-Aldrich, St. Louis, MO, USA) dissolved in 1 mL of acetone and added dropwise to 10 mL of 1.5% (w/v) polyvinyl alcohol (PVA; Sigma-Aldrich), stirring at 1000 rpm, using a syringe pump at 10 mL/hr. The nanoparticle (NP) suspension was left to stir overnight at 500 rpm to evaporate acetone. PLGA-NPs were purified by five rounds of centrifugation at 15,000×*g* and resuspension in 1 mL of DI water. Finally, the NP suspension was filtered through a 1 µm strainer to remove aggregates. Following purification, PLGA-NPs were suspended in DI water at 10 mg/mL and stored at 4 °C. For celecoxib-encapsulated PLGA-NPs (Cx-NPs), 200 mg of celecoxib (Sigma-Aldrich) was dissolved with 40 mg PLGA in 1 mL acetone. Rhodamine-encapsulated PLGA-NPs (Rhod-NPs) were prepared by adding 500 µL of rhodamine 6G (Acros Organics, Waltham, MA, USA), dissolved in ethanol at 20 mg/mL, to 40 mg PLGA in 1 mL acetone. Concentration was determined using NanoSight NS300 Nanoparticle Tracking Analysis (Malvern Panalytical, Malvern, Worcestershire, UK).

### Preparation of poly(lactic acid) hydrogel

One gram of poly(d, l-lactide)-*b*-poly(ethylene glycol)-*b*-poly(d, l-lactide) (PLA-PEG-PLA; PolySciTech, West Lafayette, IN, USA) was dissolved in 5 mL of DI water by stirring at 4 °C for 72 h to prepare 20% (w/v) solution. To prepare PLA-gel with 5% (w/v) NPs (NP/PLA-gel), 200 µL were prepared at a time by centrifuging 1 mL of NP suspension at 15,000×*g* for 10 min, followed by removal of the supernatant and resuspension in 20% PLA-gel. Rheological data of the PLA-gel is publicly available, provided by the manufacturer [28].

### Nanoparticle characterization

The average nanoparticle diameter, polydispersity index (PDI), and zeta potential were measured by dynamic light scattering (DLS) using a zeta-sizer (Malvern Panalytical). NPs were suspended in purified DI water at 5 mg/mL and filtered with a 1 µm strainer before measurements. All measurements were performed in triplicate.

### SEM imaging

SEM images were captured by Quattro ESEM (Thermo Fisher Scientific, Waltham, MA, USA). NPs were imaged using conventional methods with high vacuum following sputter coating of lyophilized NPs with iridium. For the PLA-gel, the Environmental SEM method was employed in which the samples were imaged without drying or sputter coating and remained in the solution/gel state.

### HPLC

High-performance liquid chromatography was performed using UltiMate 3000 UHPLC (Thermo Fisher Scientific) with Acquity UPLC HSS T3 column (1.8 µm, 2.1 × 150 mm; Waters, Milford, MA, USA). The samples were prepared by adding 500 µL of dichloromethane (DCM) to 500 µL Cx-NP, creating a two-phase solution, followed by vortexing for 30 s to rupture the NPs. Then, the top aqueous phase was removed and centrifuged to confirm the presence of PLGA. The DCM was evaporated in a fume hood and the remaining celecoxib was dissolved in 1 mL of a 75:25 methanol and water mixture. The extracted celecoxib samples were diluted five times with 0.1% formic acid in a 1:1 mix of water and acetonitrile, and 10 µL was loaded at a time with a flow rate of 0.30 mL/min and detection wavelength of 254 nm.

### PLGA-NP uptake

Bone marrow-derived cells (BMDCs) were freshly isolated from female B6 mice and cultured in RPMI media containing 0.2 mg/mL Rhod-NPs. After 24 hr of incubation, the cells were filtered with a 20 µm strainer, fixed with 10% buffered formalin, and adhered onto a poly-lysine-coated glass slide using StatSpin CytoFuge 2 cytocentrifuge (Beckman Coulter, Brea, CA, USA) for 10 min at 1,200 rpm then counterstained with DAPI (Vector Laboratories, Newark, CA, USA). Immunofluorescence staining was performed following incubation with Rhod-NPs for 24 hr. The cells were stained for CD3, CD45, or Ly6G with Alexa 647-labeled antibody for 1 h at RT, followed by filtration, fixation, and transfer to a glass slide. The cells were then counterstained with DAPI and imaged using a Leica DM 2500 (Leica Biosystems, Nussloch, Germany) fluorescence microscope with a 40× objective.

### Release profile of PLGA-NP from PLA-gel

Rhod-NPs were prepared and suspended at 5% in 20% PLA-gel (Rhod-NP/PLA-gel). Then, 200 µL of the Rhod-NP/PLA-gel was solidified in 1.5 mL tubes and incubated in 200 µL of 50 mM Tris–HCl buffer (pH 7.5) containing 4 µg/mL proteinase K at 37 °C (*n* = 5). PLA-gel loaded with NPs with no Rhod was prepared as a baseline control. The supernatant was collected every two days and supplemented with fresh buffer until the gel was completely degraded. The fluorescence intensity was measured using EnVision Multilabel Plate Reader (Perkin Elmer, Shelton, CT, USA) with excitation at 544 nm and emission at 579 nm. A standard curve was generated by measuring the fluorescence intensities of NP suspensions with known concentrations, ranging from 0.5 to 100 µg/mL, to determine the concentration of released NPs. NP concentration was converted to mass and plotted as the cumulative fractional release of the total Rhod-NP collected as the PLA-gel degraded.

### Cell culture

The Py230 mouse breast cancer cell line carrying mCherry reporter gene was cultured in Ham’s F-12 K (Kaighn’s) Medium (Thermo Fisher) supplemented with 5% fetal bovine serum (FBS), 1% antibiotic–antimycotic (Thermo Fisher) and 0.1% MITO + Serum Extender (Corning, NY, USA) as described previously [13]. Primary BMDCs were isolated from the bone marrow of female B6 mice and suspended in RPMI medium [29]. Following the removal of erythrocytes, the cell suspension was cultured in RPMI supplemented with 5% FBS and 1% antibiotic–antimycotic in an uncoated 3 cm dish. All cells were cultured in a humidified incubator at 37 °C, 5% CO_2_.

### Mouse model of breast cancer

C57BL/6J (B6; strain #000664) mice were purchased from The Jackson Laboratory (Bar Harbor, ME, USA) and maintained in a pathogen-free facility. Py230 cells at a density of 2 × 10^6^ cells per 100 µL in a 1:1 mix of RPMI-1640 and Matrigel GFR (Corning) were injected into the abdominal mammary fat pad of 6-week-old female B6 mice. Tumor size was measured by caliper every 3 days and volume was calculated using the formula *L* × *W*^2^ × 0.5, where *L* is the long diameter and *W* is the short perpendicular diameter. A needle biopsy was performed once tumor volume reached approximately 120 mm^3^ using a vacuum-assisted device connected to a 20-gage needle. The needle was inserted 5 mm (length equivalent to approximately half the major axis) into the tumor horizontally and slowly removed once the pressure reached -70 kPa. Immediately following needle biopsy, 2 µL of 20% PLA-gel containing 5% NPs, with no payload or loaded with celecoxib or rhodamine, was slowly injected into the biopsy cavity using a nano-syringe (Hamilton Company, Reno, NV, USA) connected to a 33-gage blunt needle to ensure injection into the biopsy cavity. Following injection, the needle was held inside the cavity for 10 sec to ensure gelation of the PLA-gel. The mice were euthanized, and the tumors were resected 1 or 15 days later. To quantify mCherry expressing breast cancer cells, the lungs were digested with 62.5 μg/mL Liberase (MilliporeSigma, Burlington, MA, USA) and 500 units/mL DNase I (MilliporeSigma). The cell suspension was incubated with a Zombie Aqua viability kit (BioLegend, San Diego, CA, USA) and analyzed using an S1000 EON benchtop flow cytometer (Stratedigm, San Jose, CA, USA). For in vivo release, the amount of Rhod-NP equal to one intra-biopsy cavity injection (0.1 mg) was suspended in 100 µL of PBS and intravenously injected via the tail vein. Tumors and organs were resected 1 and 15 days after the injection and embedded in OCT to generate frozen sections at a 6 µm thickness. The sections were counterstained with DAPI and mounted with Vectashield Antifade Mounting Medium (Vector Laboratories). Images were visualized by Leica DM 2500 (Leica Biosystems) fluorescence microscope and scanned using ZEISS Axio Scan.Z1 Digital Slide Scanner (Carl Zeiss Microscopy, Oberkochen, Germany).

### Immunohistochemistry

Immunohistochemistry was performed on FFPE sections (4 μm) as described previously [13] using antibodies listed in Supplementary Information. The slides were scanned using ZEISS Axio Scan.Z1 Digital Slide Scanner (Carl Zeiss Microscopy) with a 40× objective. Image data were acquired using ZEN lite software (Carl Zeiss Microscopy) and quantified using Image J (NIH, Bethesda, MD) with the Color Deconvolution plugin [30] (n = 10). Multi-color immunofluorescence staining was performed using FFPE sections (4 μm). Slides were incubated with a combination of the primary antibodies (CD45, CD31, E-cadherin, pan-cytokeratin (CK), and vimentin) and corresponding secondary antibodies. Slides were quenched with TrueVIEW Autofluorescence Quenching Kit (Vector Laboratories), counterstained with DAPI, and mounted with Vectashield Plus Antifade Mounting Medium (Vector Laboratories). Fluorescently stained slides were scanned using ZEISS Axio Scan.Z1 Digital Slide Scanner (Carl Zeiss Microscopy) with 40× objective. Image data were acquired using ZEN lite software (Carl Zeiss Microscopy). For quantification of E-cadherin^+^(green)/CK^+^(red) cells and vimentin^+^(red)/CK^+^(green) cells, the number of yellow pixels (green and red overlapping pixels) was counted and normalized with the number of blue pixels (nucleus) per field of view at a final magnification of 40× (*n* = 4), using Image J.

### ELISA

Freshly isolated mouse Mϕ were treated with Cx-NPs or control NPs without payload (Ctrl-NPs) at a concentration of 0.5 mg/mL in a 96-well plate for 6 h followed by incubation with or without 1% wound fluid, isolated from biopsied Py230 mouse breast tumors, for 4 h. The resulting supernatants were subjected to ELISA assay to quantify the concentration of PGE_2_ following the vendor’s instructions (Cayman Chemical, Ann Arbor, MI, USA). PGE_2_ concentration was compared using one-way ANOVA. Data is expressed as pg per mL with mean ± SD (*n* = 3).

### Wound fluid preparation

Gas-sterilized surgical sponge (Medtronic, Minneapolis, MN, USA) at a size of 7 × 5 × 2.5 mm was implanted into Py230 mouse breast tumor. The surgical sponge was collected 7 days later and centrifuged at 10,000 rpm on a spin column. The supernatants were used for assays at 1% final concentration.

### Statistical analysis

Data obtained from experiments were statistically analyzed to provide 95% power for a test’s significance level of 0.05, as depicted by *p < 0.05; **p < 0.01; ***p < 0.001; ****p < 0.0001, CI = 95%. Two group comparisons were done using the *t* test if the assumptions of normal distribution were appropriate. Otherwise, the Wilcoxon test was used. One-way ANOVA was used to compare multiple groups. All statistical analyses were performed using Prism 9 software (GraphPad Software, La Jolla, CA, USA) or R 4.1.2 (R Foundation for Statistical Computing, Vienna, Austria).

## Results

### Sustained inflammation and M2-like Mϕ accumulation in the biopsy cavity

**Fig. 1:**
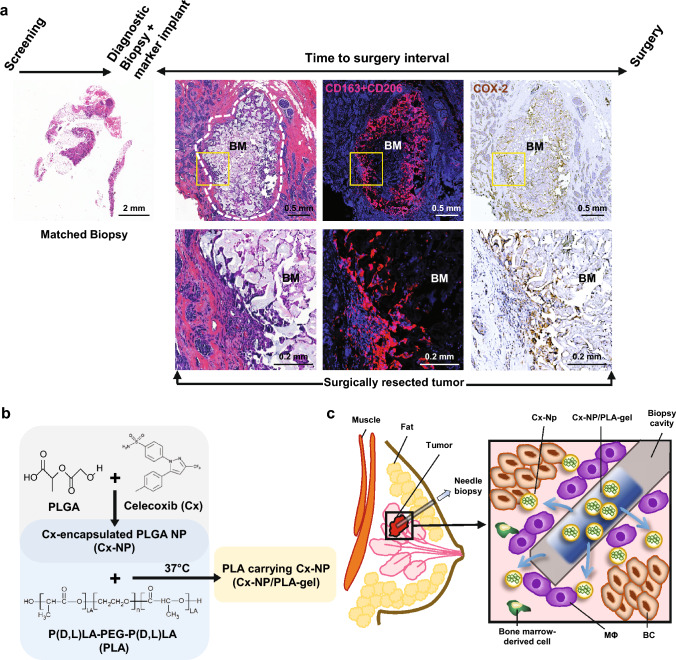
Local delivery strategy of celecoxib-encapsulated NPs for the control of protracted COX-2-associated inflammation in biopsy cavity. **a** H&E images of surgically resected Stage II breast tumor with a biopsy to surgery interval of 29 days and the matched biopsied tissue. The biopsy cavity is outlined by a white dotted line. Multiplex immunofluorescence images of CD163^+^/CD206^+^ (red) of the surgically resected tumor, counterstained by DAPI (blue). Immunohistochemical staining of COX-2 (brown), counterstained by hematoxylin (blue); BM: biopsy-site marker. **b** Experimental design of a combinatorial delivery system of thermosensitive, biodegradable PLA-hydrogel loaded with celecoxib-encapsulated PLGA-NPs. **c** Experimental design of sustained local delivery of celecoxib, encapsulated by PLGA-NPs, into BMDCs in the biopsy cavity to control protracted inflammation and cancer cell EMT

Mammographically identified suspicious lesions are generally subjected to needle biopsy for definitive diagnosis of breast malignancy. Following tissue collection, a biopsy-site marker is placed within the biopsy cavity. Figure 1 shows representative H&E images of a surgically resected stage I breast tumor 29 days following needle biopsy with a ring-shaped foreign material structure within the biopsy cavity visible at a low-power magnification (Fig. 1a and Supplementary Fig. 1). A high-power magnification image showed abundant infiltrating cells with moderate foreign body reaction around the biopsy cavity (Fig. 1a); such extensive inflammation was not detected in the corresponding matched biopsy tissue (Fig. 1a, left panel). Multiplex immunofluorescence staining of the surgically resected tumor showed a locally dense accumulation of layers of wound stroma dominated by CD163^+^/CD206^+^ M2-like Mϕ adjacent to the biopsy cavity, while M2-like Mϕ were much more sparsely distributed in the peripheral tumor stroma (Fig. 1a, Supplementary Fig. 1). Additional immunohistochemical staining indicated elevated levels of COX-2 protein expression inside and around the biopsy cavity (Fig. 1a), suggesting protracted inflammation around the biopsy wound. Since protracted inflammation with elevated COX-2 activity is a key factor underlying biopsy-associated pro-metastatic changes [13], we mimicked the clinically used biopsy-site marker to explore a novel preventive strategy in tumor-bearing mice to achieve sustained release of celecoxib into the biopsy cavity from PLA-gel loaded with Cx-NPs (Cx-NP/PLA-gel; Fig. 1b). This strategy is intended to mitigate prolonged inflammation and reduce the accumulation of M2ϕ at the biopsy site by preferential delivery to BMDCs within the surrounding wound stroma adjacent to the biopsy cavity (Fig. 1c).

### Characterization of PLGA-nanoparticles

**Fig. 2:**
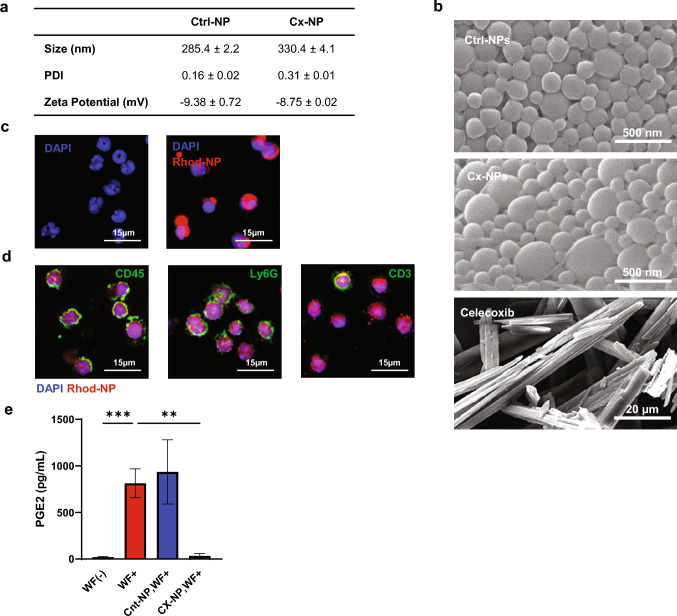
Characterization of PLGA-NP and uptake by BMDCs. **a** Table summarizing the average particle diameter, polydispersity index (PDI), and zeta potential of Cx-NPs and Ctrl-NPs. Data shown as mean ± SD (n = 3). **b** Scanning electron microscopic images of Ctrl-NPs, Cx-NPs, and crystalline celecoxib. **c** Fluorescent microscopic images of Rhod-NP uptake by mouse BMDCs. BMDCs were incubated with 2% (w/v) Rhod-NPs (red) for 24 h. BMDCs were fixed and counterstained with DAPI (blue). **d** Immunofluorescent staining images of BMDCs incubated with Rhod-NPs (red) followed by Alexa 647-labeled CD45, Ly6G, or CD3 antibodies. Fluorescence microscopic images were captured at a final magnification of 40×. The red color represents the rhodamine signal captured in the red channel; the green represents the digitally processed pseudo-color of far-red fluorescence. **e** PGE_2_ quantification by ELISA of Mϕ incubated with Cx-NPs or Ctrl-NPs for 6 h. Following incubation with NPs, Mϕ were treated with or without 1% wound fluid collected from biopsied Py230 breast tumor. Data is expressed as pg/mL with mean ± SD (*n* = 3) and analyzed using one-way ANOVA

Analysis of the physicochemical properties of Cx-NPs and Ctrl-NPs showed average particle sizes of 330.4 ± 4.1 nm and 285.4 ± 2.2 nm, respectively (Fig. 2a, Supplementary Fig. 2a). The PDI of Cx-NPs and Ctrl-NPs was 0.31 ± 0.01 and 0.16 ± 0.02, respectively, while the corresponding zeta potentials were − 8.75 ± 0.02 mV and − 9.38 ± 0.72 mV, within the neutral charge range (Fig. 2a**, **Supplementary Fig. 2b). The slightly larger particle size with broader size distribution for Cx-NPs than Ctrl-NPs was presumably due to the hydrophobic payload. The amount of celecoxib encapsulated by PLGA-NPs was quantified by reverse phase HPLC and UV detection at 254 nm following liquid–liquid extraction by DCM (Supplementary Fig. 3). Celecoxib concentration was estimated at 31.25 µg/mL NPs (5 mg/mL PLGA-NP). SEM images of crystalline solid celecoxib revealed needle- and rod-shaped structures of varying widths and lengths within the micrometer size range. Both Cx-NPs and Ctrl-NPs were significantly smaller in size, in the nanometer range, and displayed a smooth and spherical morphology (Fig. 2b).

Next, we examined the uptake of Rhod-NPs by freshly isolated mouse BMDCs. After 24 h of incubation, fluorescence microscopic analysis showed substantial uptake of Rhod-NPs into a range of BMDCs (Fig. 2c). Direct immunostaining with fluorophore-labeled CD45 antibody confirmed the uptake of Rhod-NPs by both CD45^+^ (Fig. 2d) and CD45^−^ BMDCs (Supplementary Fig. 4a), suggesting uptake by both hematopoietic and non-hematopoietic BMDCs. To evaluate the uptake of Rhod-NPs by BMDC types, further staining with the lymphocyte marker CD3 or myeloid cell marker Ly6G was performed, confirming uptake by both cell types (Fig. 2d and Supplementary Fig. 4b). ELISA assay confirmed Cx-NP inhibitory effect of COX-2 activity by reduction of PGE_2_ release from Mϕ, treated with 1% wound fluid, compared to Ctrl-NP-treated Mϕ (Fig. 2e).

### Sustained payload release from biodegradable PLA-gel in vitro

**Fig. 3:**
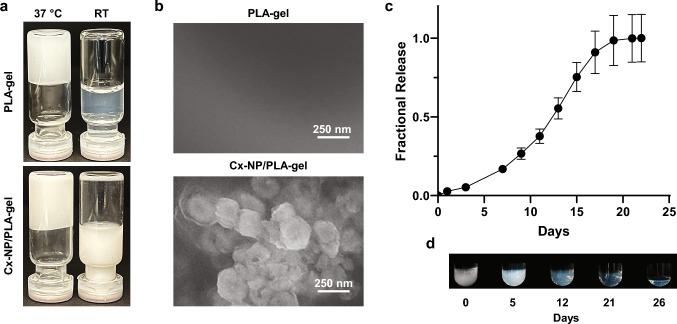
Characterization of NP/PLA-gel. **a** Inversion test of 20% (w/v) PLA-gel and Cx-NP/PLA-gel (5% w/v NP) at 37 °C and RT. **b** Environmental scanning electron microscopic images of PLA-gel alone or PLA-gel loaded with Cx-NPs. **c** Release profile of Rhod-NPs from PLA-gel. Following solidification, Rhod-NP/PLA-gels were incubated in digestion buffer (50 mM Tris–HCl buffer at pH 7.5 containing 4 µg/mL proteinase K) at 37 °C for 22 days. The supernatants were collected at indicated time points. The graph depicts cumulative fractional payload release. Data shown as mean ± SD (*n* = 5). **d** Photographic image of time-dependent degradation of PLA-gel at 37 °C. The solidified PLA-gel was incubated in the digestion buffer at 37 °C and images were taken at indicated time points

An inversion test for thermosensitive properties of the PLA-gel and Cx-NP/PLA-gel (5% w/v NP) at a final concentration of 20% (w/v) showed the ability for rapid solidification and retention of a solid state at 37 °C, while remaining a viscous solution at room temperature. Notably, the inclusion of NPs did not affect the thermosensitive gelation capacity of the PLA-gel at 37 °C (Fig. 3a). PLA-gels at 15% (w/v) and lower concentrations remained as a semi-solid slurry (Supplementary Fig. 5). Thus, PLA-gel at 20% (w/v) was used for the study. SEM images of Cx-NP/PLA-gel showed spherical particles, presumably Cx-NPs, equivalent in size to a single NP, distributed within a sheet-like structure of PLA-gel, whereas PLA-gel alone was featureless due to absence of NPs (Fig. 3b). To examine the payload release pattern, solidified Rhod-NP/PLA-gel (20% w/v) was incubated in a buffer at 37 °C. Red fluorescent intensity (i.e., rhodamine) in the supernatant was measured over three weeks. The cumulative release profile displayed a slow release of Rhod-NPs in the first 5 days, followed by an accelerating linear release for 15 days, until plateauing at approximately day 20 (Fig. 3c). Corresponding photographic images showed a gradual degradation of PLA-gel at 37 °C based on increased transparency and a decrease in the overall size of the PLA-gel over time (Fig. 3d).

### Sustained local release of Rhod-NPs from PLA-gel injected into the biopsy cavity of mouse tumors

**Fig. 4:**
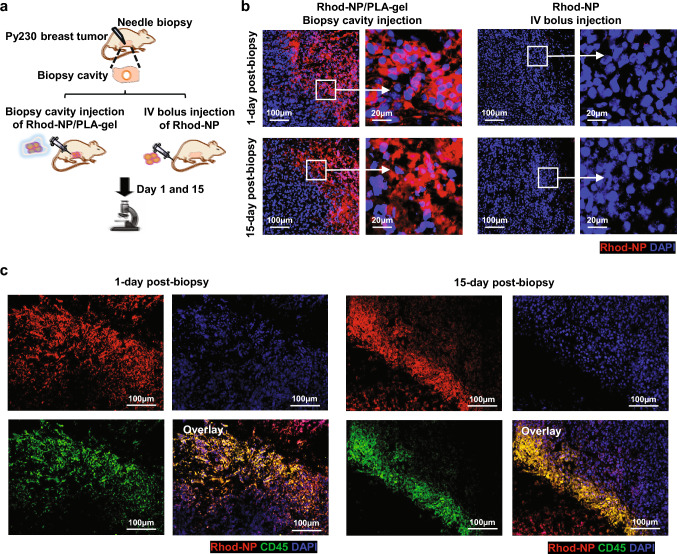
Sustained release of Rhod-NPs and uptake by BMDCs following intra-biopsy cavity injection of Rhod-NP/PLA-gel. **a** Schematic depicting experimental design for intra-biopsy cavity injection of Rhod-NP/PLA-gel and IV bolus injection of Rhod-NPs. Tumors and organs of reticuloendothelial system were resected 1 and 15 days after the injection. **b** Cellular uptake of Rhod-NPs in the biopsy cavity of Py230 tumors 1 and 15 days after the intra-biopsy cavity injection of Rhod-NP/PLA-gel or tail vein injection of Rhod-NPs. The frozen sections were counterstained by DAPI (blue). The images were captured using Zeiss AxioScan with a 40× objective. **c** Uptake of Rhod-NPs by CD45^+^ cells around the biopsy cavity of Py230 tumors. Frozen sections were immunostained with Alexa 647-labeled CD45 antibody (green) and counterstained with DAPI. The images were captured and processed using Zeiss AxioScan with a 40× objective

Based on the thermosensitive solidification, sustained release of NPs from PLA-gel, and effective uptake of PLGA-NPs by BMDCs, we next examined the feasibility of sustained NP uptake by BMDCs in the surrounding wound stroma adjacent to the biopsy cavity, where PLA-gel is injected, using a mouse model of breast cancer. Mice bearing solitary Py230 breast tumors of approximately 120 mm^3^ in size received a single needle biopsy, followed by a single injection of Rhod-NP/PLA-gel into the biopsy cavity. The tumors were resected post-biopsy day 1 and 15 (Fig. 4a). Microscopic analysis of frozen sections revealed the presence of red fluorescence inside and around the biopsy cavity on post-biopsy day 1, which sustained until day 15 (Fig. 4b). High-power magnification images confirmed the cellular uptake of Rhod-NPs inside and at the perimeter of the biopsy cavity. In comparison, only minor red fluorescence was present on day 1 and day 15 in the biopsy cavity of mice that received a bolus intravenous injection of Rhod-NPs (Fig. 4b). Direct immunofluorescence staining with Alexa 647-labeled CD45 antibody showed a significant overlay with red fluorescent cells at the perimeter of the biopsy cavity at post-biopsy day 1 and 15 in mice that received intra-biopsy cavity injection of Rhod-NP/PLA-gel (Fig. 4c), suggesting NP uptake into CD45^+^ infiltrating immune cells, presumably due to their spatial proximity to the biopsy site. Further microscopic analysis of organs showed no apparent red fluorescence signals present in the reticuloendothelial organs (lung, liver, and spleen) or kidneys in mice that received intra-biopsy cavity injection of Rhod-NP/PLA-gel (Supplementary Fig. 6), suggesting minimal to no leakage of Rhod-NPs into the circulation from the biopsy cavity. In contrast, red fluorescence speckles were ubiquitously detected in the lungs and kidneys on day 1, which remained, albeit lesser, until day 15 in mice that received a bolus IV injection of Rhod-NP (Supplementary Fig. 6). Together, these data suggested the feasibility of sustained, preferential delivery of NPs into CD45^+^ BMDCs via local injection of a combinatorial delivery system—thermosensitive, biodegradable PLA-gel loaded with PLGA-NPs—into the biopsy cavity.

### Local injection of Cx-NP/PLA-gel into the biopsy cavity suppresses biopsy-induced metastasis

**Fig. 5:**
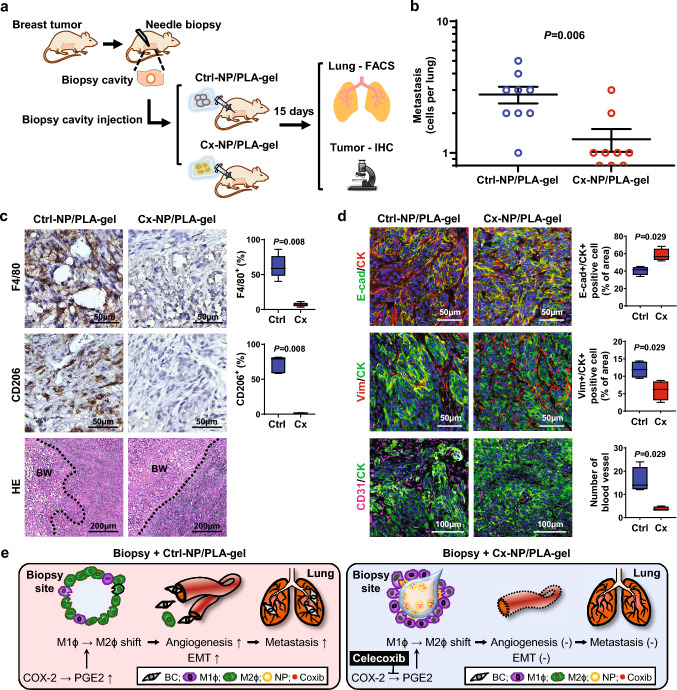
Local delivery of Cx-NP/PLA-gel decreases biopsy-induced pro-metastatic changes and pulmonary metastasis **a** Schematic depicting experimental design for intra-biopsy cavity injection of Cx-NP/PLA-gel or Ctrl-NP/PLA-gel. **b** Graph displaying the number of metastatic Py230 cancer cells in the lungs of mice 15 days after the intra-biopsy cavity injection of Cx-NP/PLA-gel or Ctrl-NP/PLA-gel. **c** FFPE sections derived from tumors that received Cx-NP/PLA-gel or Ctrl-NP/PLA-gel and immunohistochemically stained for F4/80 or CD206 (brown) and counterstained with hematoxylin (blue). Graphs summarize the densities of F4/80^+^ and CD206^+^ cells normalized by nucleus count per field of view at 40× magnification (*n* = 10). Representative H&E images are shown; BW: biopsy wound; *p*-values were calculated using the Wilcoxon test. **d** Representative images of Py230 tumors at post-biopsy day 15, injected with Cx-NP/PLA-gel or Ctrl-NP/PLA-gel, immunofluorescently stained for CK (red) and E-cadherin (green) in the top panel, CK (green) and vimentin (red) in the middle panel, and CD31 (pink) and CK (green) in the bottom panel. The slides were counterstained with DAPI (blue). Graphs summarize the percentage of E-cadherin^+^/CK^+^ cells, vimentin^+^/CK^+^ cells, or CD31^+^ blood vessel count, normalized by nucleus pixel count per field of view at a final magnification of 40× (*n* = 4). The data are shown as standard boxplots; *p*-values were calculated using the Wilcoxon test relative to Ctrl-NP/PLA-gel injected Py230 tumors. **e** Schematic image depicting a working model of intra-biopsy cavity delivery of Cx-NPs via the PLA-gel based combinatorial delivery system for preventing needle biopsy-associated pro-metastatic progression

Based on the feasibility of sustained local delivery of NPs into surrounding wound stroma adjacent to the biopsy cavity, we next examined whether sustained local delivery of Cx-NPs from biodegradable PLA-gel in the biopsy cavity reduces biopsy-associated breast cancer metastasis. Mice bearing solitary Py230 tumors carrying an mCherry reporter gene received a needle biopsy followed by a single injection of Cx-NP/PLA-gel or Ctrl-NP-loaded PLA-gel (Ctrl-NP/PLA-gel) into the biopsy cavity. Fifteen days later, the lungs and tumors were resected for the quantification of disseminated cancer cells as well as histologic analysis (Fig. 5a). A single intra-biopsy cavity injection of Cx-NP/PLA-gel significantly reduced the number of disseminated Py230^mCherry^ cells in the lungs compared to the mice that received Ctrl-NP/PLA-gel (Fig. 5b), with no notable differences in tumor growth rate or body weight (Supplementary Fig. 7). Immunohistochemistry analysis of the tumor sections showed a ninefold lower density of F4/80^+^ Mϕ (a common Mϕ marker) in the biopsy cavity of mice that received Cx-NP/PLA-gel compared to Ctrl-NP/PLA-gel (6.7 vs. 58.8, 95% CI 3.5–11.5 vs. 40.3–86.2; *p* = 0.008). Additionally, CD206^+^ M2-like Mϕ showed 43-fold lower density in mice that received Cx-NP/PLA-gel in the biopsy cavity, compared to Ctrl-NP/PLA-gel (1.8 vs. 79.2, 95% CI 0.6–2.1 vs. 58.4–81.6; *p* = 0.008; Fig. 5c).

Further multi-color immunofluorescence was performed to evaluate the extent of cancer cell EMT adjacent to the biopsy wound stroma. The expression of epithelial cell marker E-cadherin and mesenchymal marker vimentin were assessed in CK^+^ cancer cells. The loss or reduced expression of E-cadherin and gain of vimentin expression along with acquisition of mesenchymal morphology collectively indicate EMT, a sign of acquired migratory capacity (i.e., invasion) [31]. Analysis of Py230 tumors injected with Cx-NP/PLA-gel revealed a 1.4-fold increase in E-cadherin^+^/CK^+^ cancer cells compared to Ctrl-NP/PLA-gel (56.6 vs. 41.6, 95% CI 51.8–68.4 vs. 33.9–45.4; *p* = 0.029) and 1.9-fold decrease in vimentin^+^/CK^+^ cells in Cx-NP/PLA-gel injected tumors, compared to biopsied tumors injected with Ctrl-NP/PLA-gel (6.2 vs. 11.9, 95% CI 2.5–8.8 vs. 9.4–14.4; *p* = 0.029; Fig. 5d**, **Supplementary Fig. 8), suggesting a significant reduction in the level of cancer cell EMT. In parallel, CD31^+^ blood vessels were fourfold less prevalent in tumors that received Cx-NP/PLA-gel than Ctrl-NP/PLA-gel (3.5 vs. 14.0, 95% CI 3.0–5.0 vs. 12.0–24.0; *p* = 0.029; Fig. 5d, Supplementary Fig. 8). These data suggest that a single injection of Cx-NP/PLA-gel into the biopsy cavity effectively reduced biopsy-associated cancer metastasis by reducing M2-like Mϕ-mediated pro-metastatic changes such as EMT and angiogenesis (Fig. 5e).

## Discussion

Mounting evidence highlights the importance of prompt initiation of treatment, either surgery or therapy, following breast cancer diagnosis [5–9]. We have previously reported an increased risk of disease progression and mortality [13, 32] associated with delay of surgery or adjuvant treatment by just 60 days after diagnostic needle biopsy. We also recently showed that protracted COX-2 activity in the wound stroma following needle biopsy of breast tumors accelerates pro-metastatic changes via substantial accumulation of infiltrating M2-like Mϕ and their paracrine factors, effects that were mitigated by oral administration of celecoxib in a syngeneic mouse model of breast cancer [13]. Extending these previous findings, the current study demonstrated the feasibility of sustained, preferential delivery of Cx-NPs into BMDCs in the wound stroma from locally injected biodegradable PLA-gel inside the biopsy cavity to mitigate biopsy-related pro-metastatic changes.

Local delivery of therapeutic payloads offers significant advantages, most notably an increase in the therapeutic index by maintaining drug levels within a desired range through sustained release and decreased drug toxicity, thus, allowing for a reduced dosing schedule and improved patient compliance and quality of life. For example, a biodegradable PLGA hydrogel formulation of the gonadotropin-releasing hormone agonist leuprolide acetate (i.e., LupronDepot®) has been safely administered to prostate cancer patients for over 20 years, and the sustained local delivery of leuprolide has significantly improved patients’ quality of life by reducing dosing frequency [33, 34]. In the context of local delivery for cancer treatment, a few clinical trials are currently underway [35, 36]. Hydrogel microspheres loaded with irinotecan are delivered via chemoembolization directly to the liver, allowing for continuous release and more effective penetration of the tumor tissue in patients with liver metastases of colorectal origin [37]. Delivery of therapeutic payload to a tumor is traditionally achieved by molecularly targeted drug delivery or passive targeting via the enhanced permeation and retention effect [38–40]. Consistent with the literature [41, 42], we found that distribution of intravenously injected Rhod-NPs is limited primarily to the periphery of the tumor and is rarely seen within the biopsy cavity, perhaps due to damages to the vascular network caused by needle biopsy, which may not recover until the late phase of wound healing when the angiogenic switch occurs [43]. One notable advantage of the current mode of drug delivery is a reduction in dose compared to our previous report using orally administered celecoxib [13]; a single intra-biopsy cavity injection of Cx-NP/PLA-gel achieved sustained effect over the 15-day study period with a 10000-fold lower dose of celecoxib (1.45 µg celecoxib per a single intra-biopsy cavity injection of Cx-NP/PLA-gel vs. oral celecoxib dose of 45.1 mg/kg/day [13]). Generally, a high oral dose of celecoxib is required to achieve therapy primarily due to its limited bioavailability [44], poor water solubility [45], and rapid metabolism into pharmacologically inactive forms [46–48]. However, the lowest effective dose for the shortest duration necessary is preferred due to the increased risk of cardiac and renal toxicity [49] associated with long-term use of oral celecoxib, as stated in its black box warning [50]. As such, we limited the amount of celecoxib, encapsulated by NPs, to as little as 14.4 µg/mg NP, which sufficiently controlled local inflammation via sustained release over 15 days. Therefore, a locally injected combinatorial delivery system capable of sustained release may pave the way for such drugs with poor safety profiles to be repurposed.

Due to the technical challenge of injecting a solid material into the small biopsy cavity of a mouse tumor, this study adopted a thermosensitive tri-block co-polymer hydrogel, which allowed for an injection of as little as 2 µL, at a concentration of 20%, directly into the biopsy cavity at ambient temperature followed by rapid solidification at body temperature. Thermosensitive reversion of the solid PLA-gel to liquid state at room temperature, presumably due to temperature drop during processing, however, precluded histologic localization of injected PLA-gel. While dislocation of biopsy-site marker to adjacent normal tissue has been reported in clinical cases [51], partial filling or the effect on adjacent normal tissues were not evaluated in this study. The use of transgenic mouse models with established adjacent stroma and normal tissues, instead of grafting model used in this study, would better address possible differences in therapeutic effect in carcinoma vs. adjacent tissues. While we used a tri-block co-polymer with a target degradation duration of three weeks due to the biological endpoint of the mouse model used in this study, the speed and overall course of release can be further optimized by adjusting the type of carrier material, hydrogel, and NP concentration. Currently, the coating materials of clinically used biopsy site markers remain unexplored as a drug delivery carrier, providing a promising opportunity for local delivery of a therapeutic payload directly into the biopsy cavity.

In conclusion, this study provides evidence for sustained, local delivery of therapeutic payloads preferentially to BMDCs in the biopsy wound via a single injection of a combinatorial delivery system—biodegradable hydrogel loaded with celecoxib-encapsulated NPs—enabling a significant reduction of biopsy-related pro-metastatic changes and breast cancer metastasis in mice, while minimizing systemic off-target effects.

## Supplementary Information

Below is the link to the electronic supplementary material.

Supplementary file1 (PDF 4358 KB)

## Data Availability

The data generated in this study are available upon request from the corresponding author.
