# Supplementary material for: Sustained delivery of celecoxib from nanoparticles embedded in hydrogel injected into the biopsy cavity to prevent biopsy-induced breast cancer metastasis
Source: Breast Cancer Res Treat. 2024 Jul 5;208(1):165–77. doi: 10.1007/s10549-024-07410-x (PMC11452511; doi:10.1007/s10549-024-07410-x)
Supplement: Supplementary file 1 — Supplementary file1 (PDF 4358 KB) [file 10549_2024_7410_MOESM1_ESM.pdf]

Supplementary Information

Sustained Delivery of Celecoxib from Nanoparticles Embedded in Hydrogel Injected into the Biopsy Cavity to Prevent Biopsy-Induced Breast Cancer Metastasis

Reese Simmons, Hiroyasu Kameyama, Seiko Kubota, Yunguang Sun, John F. Langenheim, Rana Ajeeb, Tristan S. Shao, Samantha Ricketts, Anand C. Annan, Natalie Stratemeier, Sophie J. Williams, John R. Clegg, Kar-Ming Fung, Inna Chervoneva, Hallgeir Rui, and Takemi Tanaka

Breast Cancer Research and Treatment

Lead Contact: Takemi Tanaka, Ph.D., Professor
University of Oklahoma Health Sciences Center, School of Medicine, Department of Pathology,
Stephenson Cancer Center, 975 NE 10th St, BRC-W, Rm 1415, Oklahoma City, OK 73104
Phone: Office (405) 271-8260, Email: takemi-tanaka@ouhsc.edu

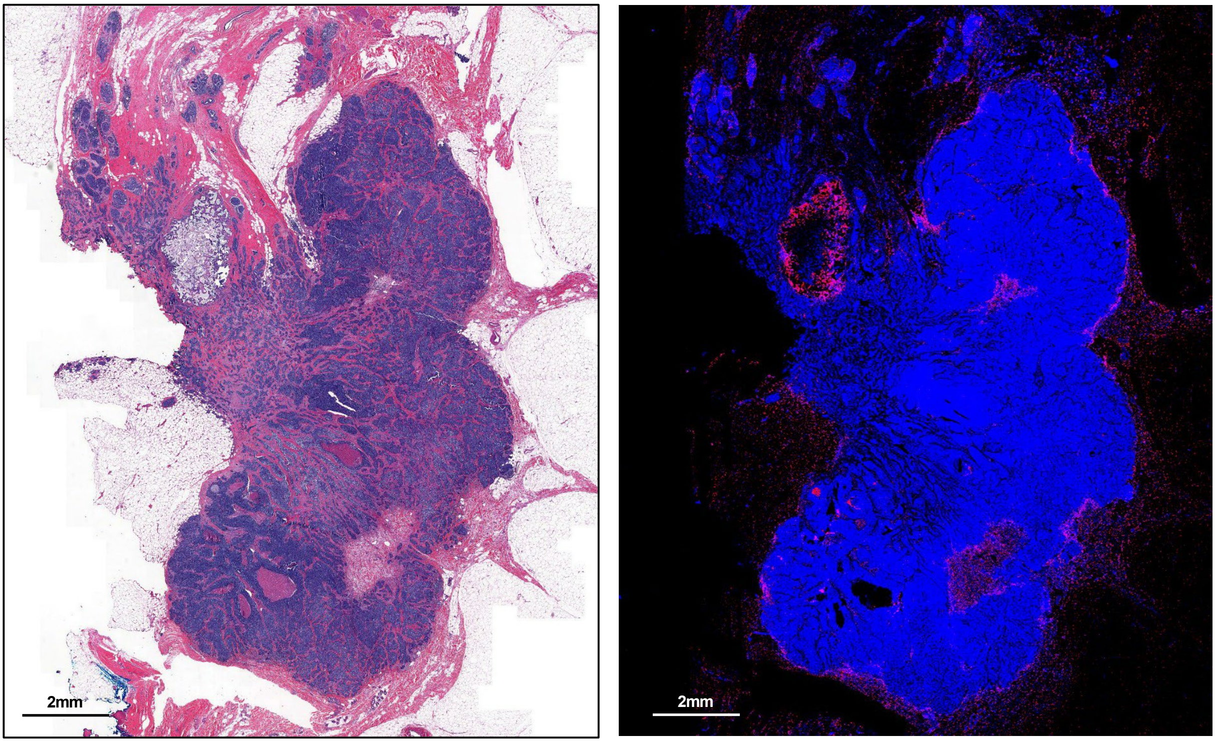

Supplementary Fig. 1: Whole mount images of surgically resected breast tumor shown in Fig. 1a. Tissue stained with H&E (left) and CD163/CD206 in red and DAPI (right).

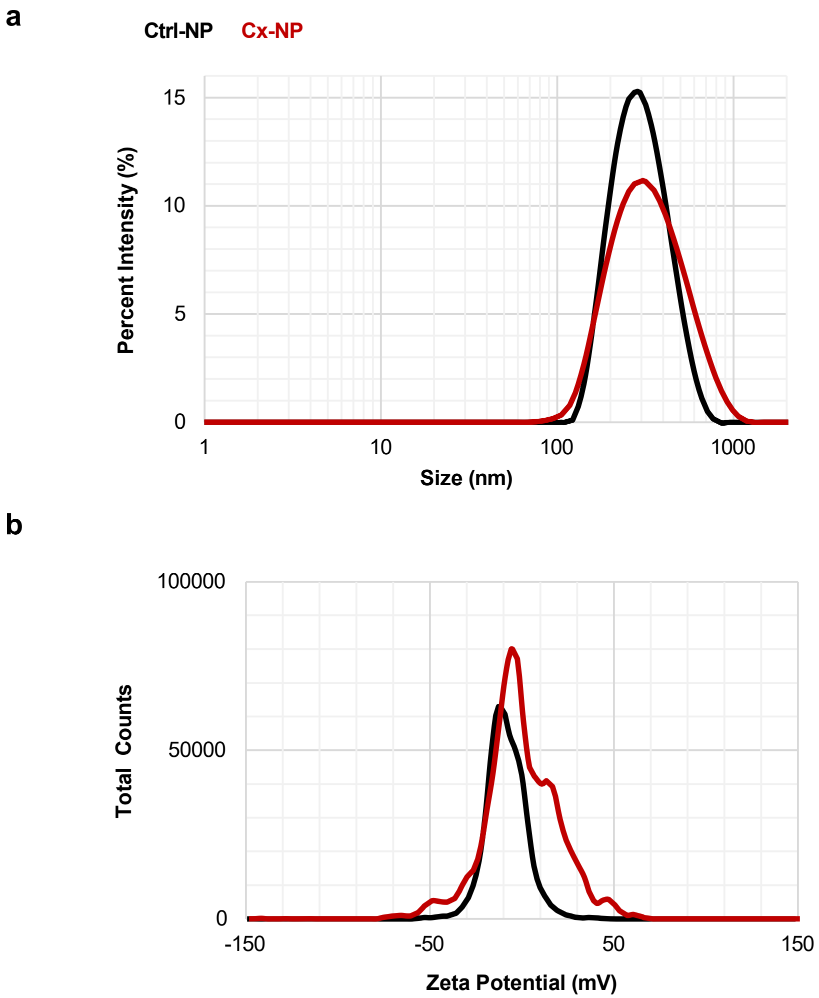

Supplementary Fig. 2: Nanoparticle size and zeta potential. (**a**) Particle size distribution and (**b**) zeta potential distribution of Cx-NPs (red) and Ctrl-NPs (black) from measurements collected by DLS analysis (n=3).

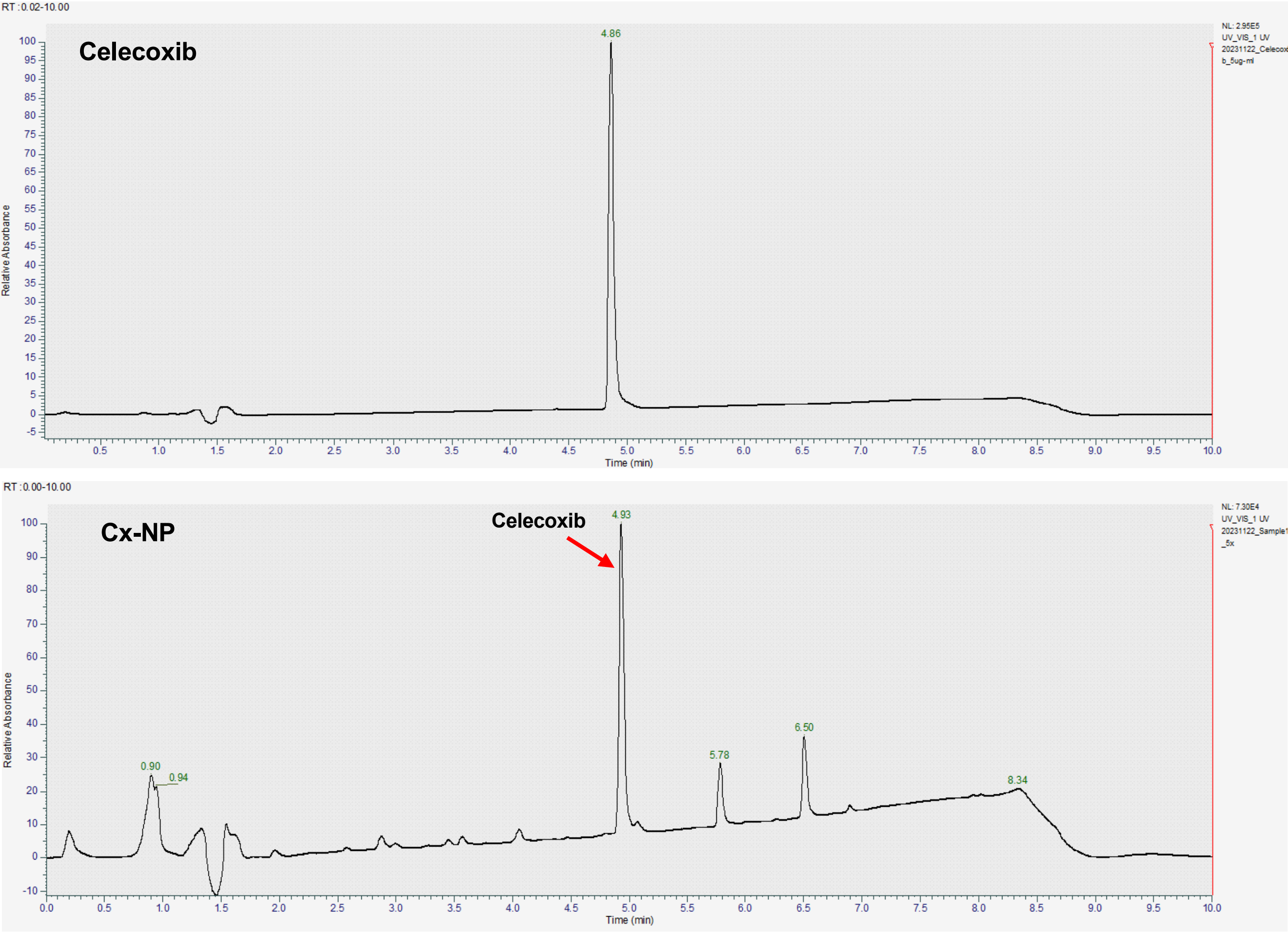

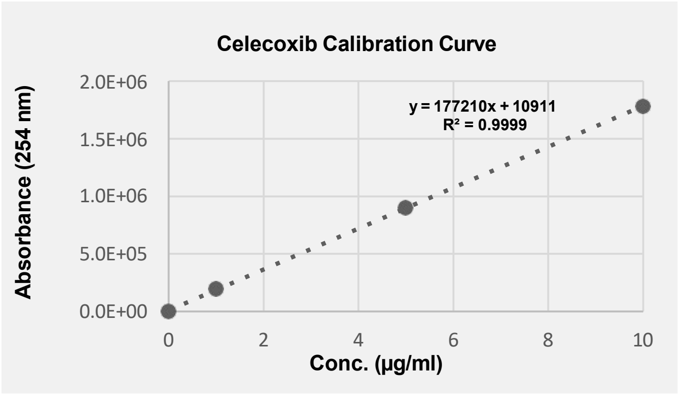

Celecoxib	Absorbance (254 nm)	Conc. (μg/mL)
#1	2.12E+05	5.7
#2	2.46E+05	6.6
#3	2.53E+05	6.8
Avg.	2.37E+05	6.38

Supplementary Fig. 3: Concentration of celecoxib encapsulated in Cx-NPs. Chromatograms of pure celecoxib (top) and celecoxib extracted from NPs (bottom) following HPLC using C18 column and UV detection at 254 nm. The table summarizes the absorption and concentration of celecoxib extracted from NPs calculated using the calibration curve.

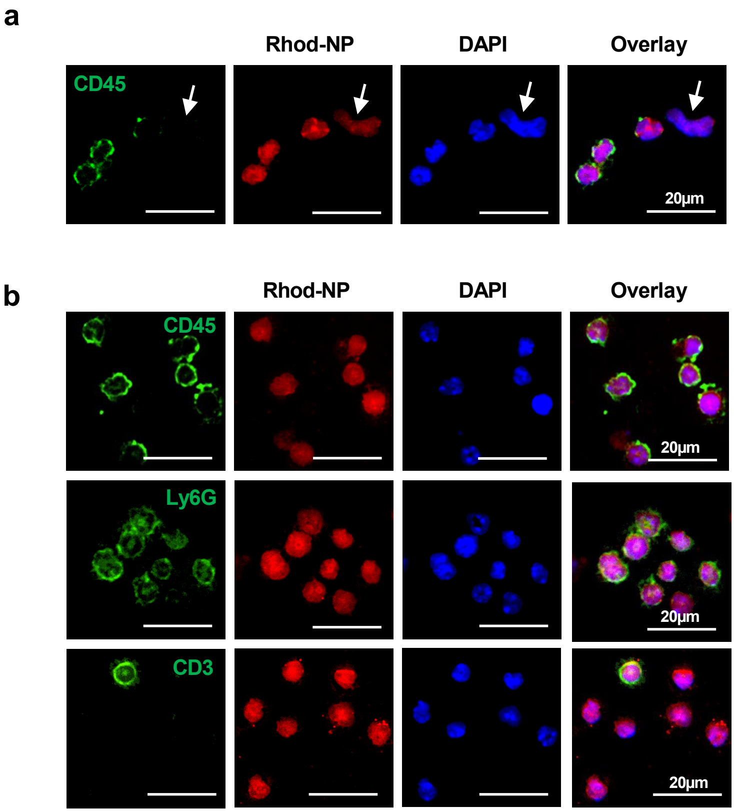

Supplementary Fig. 4: Rhod-NP uptake by BMDCs. (**a-b**) Single color and overlay images of BMDCs incubated with Rhod-NPs (red) for 24 hours, stained for CD45, Ly6G, or CD3 (green), and counterstained with DAPI (blue). (**a**) The white arrow indicates CD45-negative BMDCs with positive Rhod-NP uptake. Images were captured using a Leica fluorescent microscope with a 40x objective. Scale bar = 20 μm.

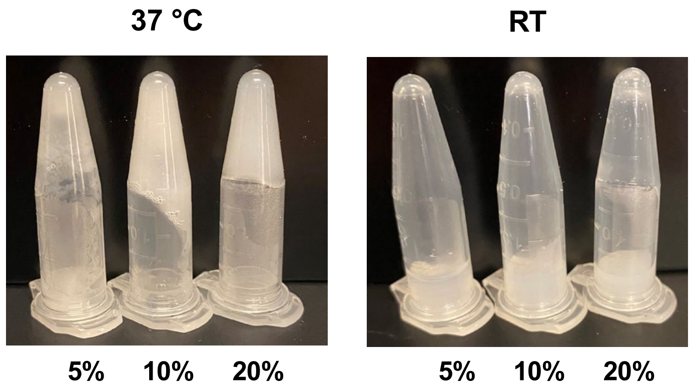

Supplementary Fig. 5: Effect of concentration on gelation capacity of PLA-gel. Inversion test of 5%, 10%, and 20% PLA-gel at 37 °C and room temperature.

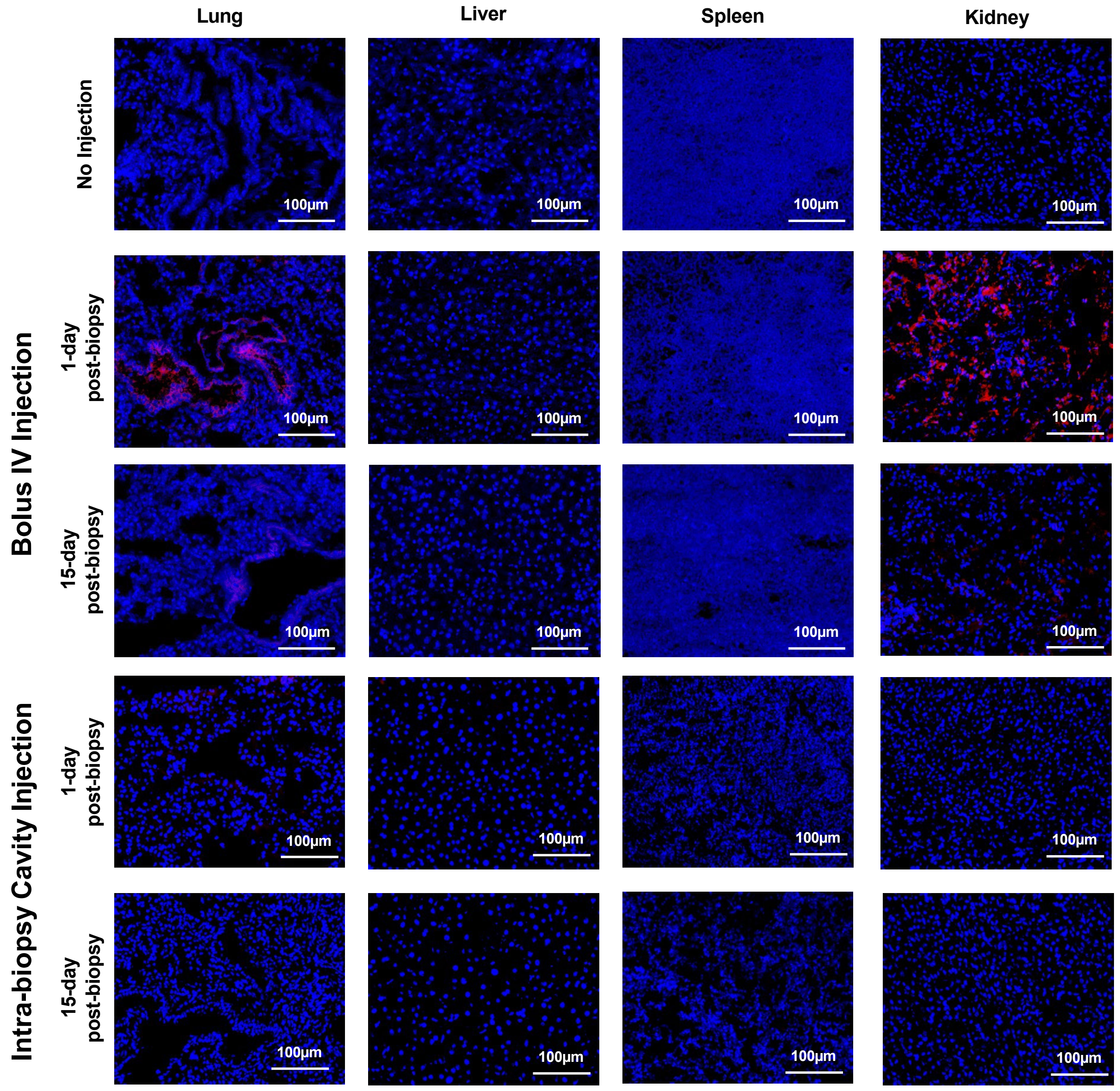

Supplementary Fig. 6: Rhod-NP in RES organs and kidneys following bolus IV injection compared to intra-biopsy cavity injection of Rhod-NP/PLA-gel. Representative images of RES organs (lung, liver, spleen) and kidneys of mice that received either no injection, a single IV bolus injection of Rhod-NP, or intra-biopsy cavity injection of Rhod-NP/PLA-gel. Frozen sections were counterstained with DAPI (blue). Images were acquired microscopically using a Leica fluorescent microscope with a 20x objective.

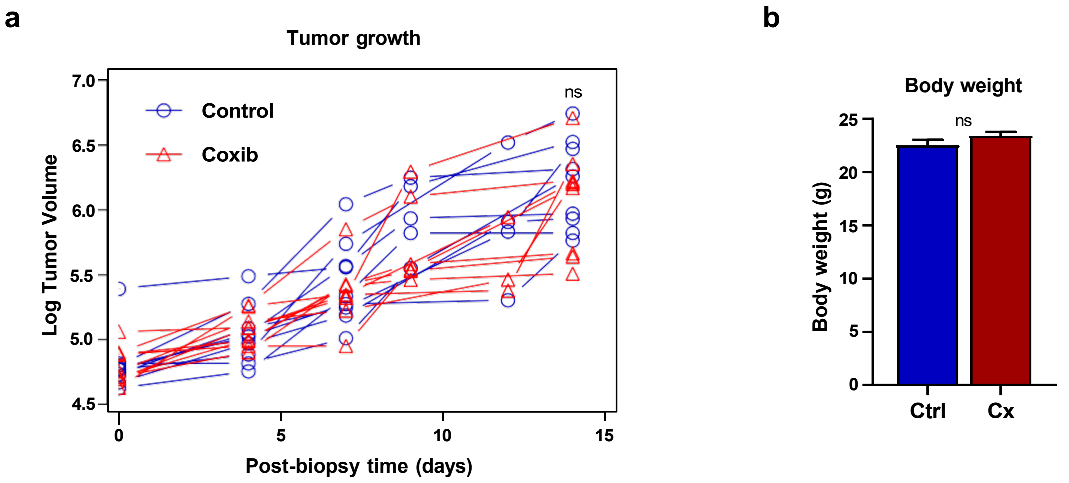

Supplementary Fig. 7: No significant difference in tumor growth or body weight between mice that received intra-biopsy cavity injection of Cx-NP/PLA-gel or Ctrl-NP/PLA-gel. (**a**) Spaghetti plot depicting tumor growth throughout the 15-day study period of individual mice assigned to the study. (**b**) Body weight at the time of study endpoint in mice assigned to the study.

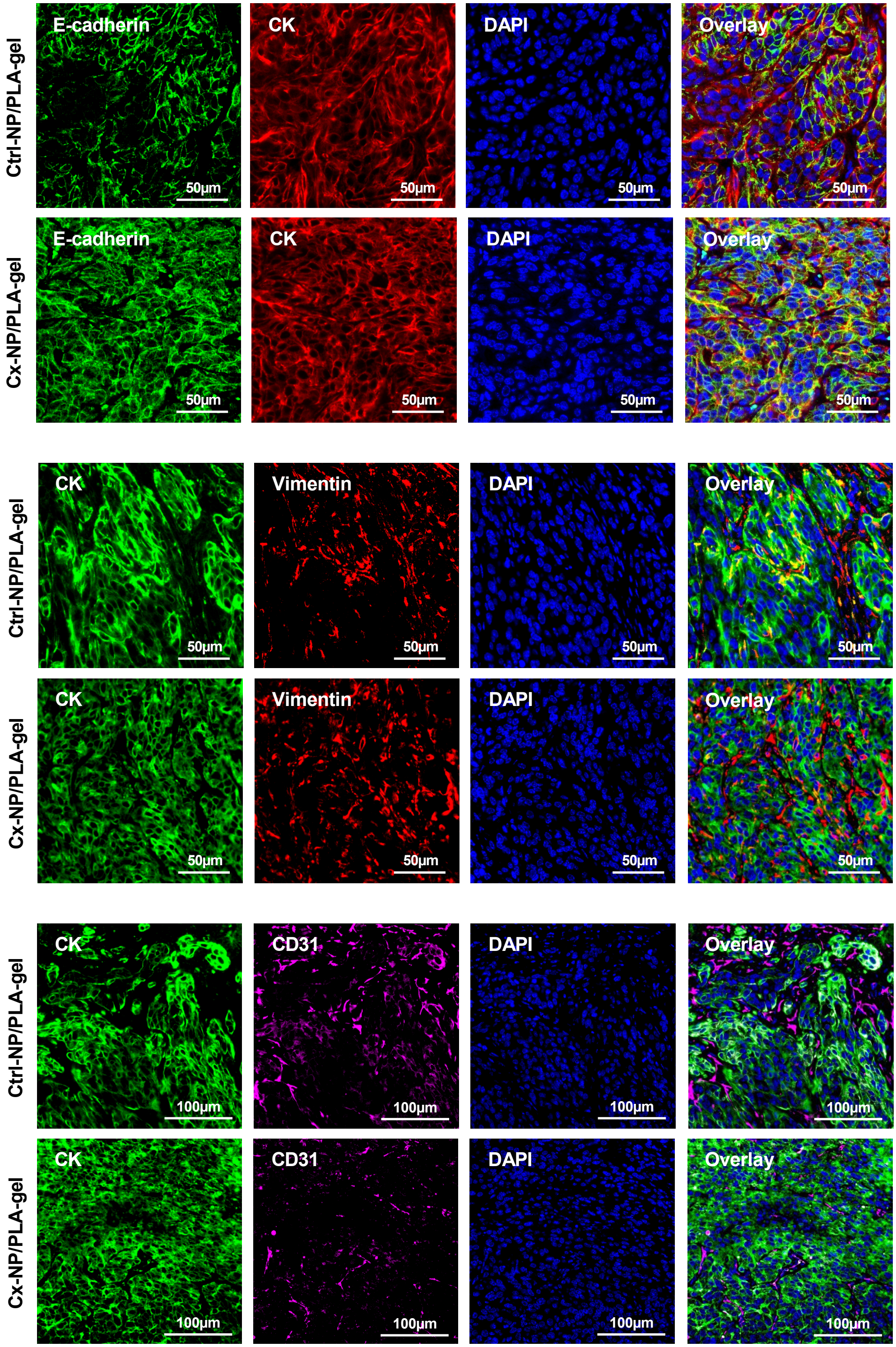

Supplementary Fig. 8: Single color and overlay images of immunofluorescence staining of Py230 tumors that received Ctrl-NP/PLA-gel or Cx-NP/PLA-gel shown in Fig. 5d. E-cadherin (green) and CK (red) are shown in the top panel, CK (green) and vimentin (red) in the middle panel, CK (green) and CD31 (pink) are shown in the bottom panel with DAPI (blue) and overlay of all colors for each panel. Images were acquired using Zeiss AxioScan with a 40x objective.

Key Resources Table

Antibody	Source	Identifier
CD206	Abcam	ab64693
F4/80	Genetex	GTX-26640
E-cadherin	Thermo Fisher Scientific	13-1900
Pan-Cytokeratin	Abcam	ab6401
Pan-Cytokeratin	Dako	M3515
Vimentin	Thermo Fisher Scientific	PA5-27231
CD31	R&D Systems	AF3628
COX-2	Biocare Medical	CRM306A
mCherry	Thermo Fisher Scientific	PA5-34974
MACH 2 Mouse HRP-Polymer	Biocare Medical	MHRP520
MACH 2 Rabbit HRP-Polymer	Biocare Medical	RHRP520
Rat-on-Mouse HRP Polymer	Biocare Medical	RT517
Anti-rat Alexa Fluor Plus 488	Thermo Fisher Scientific	A48269
Anti-rabbit Alexa Fluor Plus 555	Thermo Fisher Scientific	A32732
Anti-mouse Alexa Fluor Plus 647	Thermo Fisher Scientific	A32728TR
Anti-goat Alexa Fluor Plus 488	Thermo Fisher Scientific	A32814
Anti-mouse Alexa Fluor 568	Thermo Fisher Scientific	A11031
Anti-rat Alexa Fluor Plus 647	Thermo Fisher Scientific	A48265
Alexa Fluor 647-CD3	BioLegend	100209
Alexa Fluor 647-CD45	BioLegend	103123
Alexa Fluor 647-Ly6G	BioLegend	127609
FITC-CD45	eBioscience	11-9459-42

Material	Source	Identifier
Resomer RG 502H, Poly(D,L-lactide-*co*-glycolide)	Sigma-Aldrich	719897
Poly(D,L-lactide)-*b*-Poly(ethylene glycol)-*b*-Poly(D,L-lactide)	PolySciTech	AK100
Celecoxib	Sigma-Aldrich	PHR1683
Rhodamine 6G	Acros Organics	191410500
Proteinase K	Bio Basic	PB0451-50
Matrigel GFR	Corning	356231
DNase I	MilliporeSigma	10104159001
Liberase	MilliporeSigma	05401119001
Zombie Aqua Fixable Viability Kit	BioLegend	423102
Betazoid DAB Chromogen	Biocare Medical	BDB2004
TrueVIEW Autofluorescence Quenching Kit	Vector Laboratories	SP-8500-15
100 µL Gastight Luer Lock Syringe Model 1710	Hamilton Company	81020
Portable Phlegm Suction Unit	N/A	H003-B
PGE_2 ELISA Kit	Cayman Chemical	514010
Surgical Sponge	Medtronic	CF100

Experimental Models	Source	Identifier
Py230 murine breast cancer cell line	Gift from Dr. Lesley G. Ellies (University of California, San Diego, CA, USA)	N/A
C57BL/6J (B6) mice	The Jackson Laboratory	Strain #: 000664
